# *REFMAC*5 for the refinement of macromolecular crystal structures

**DOI:** 10.1107/S0907444911001314

**Published:** 2011-03-18

**Authors:** Garib N. Murshudov, Pavol Skubák, Andrey A. Lebedev, Navraj S. Pannu, Roberto A. Steiner, Robert A. Nicholls, Martyn D. Winn, Fei Long, Alexei A. Vagin

**Affiliations:** a Structural Biology Laboratory, Department of Chemistry, University of York, Heslington, York YO10 5YW, England; b Biophysical Structural Chemistry, Leiden University, PO Box 9502, 2300 RA Leiden, The Netherlands; c Randall Division of Cell and Molecular Biophysics, New Hunt’s House, King’s College London, London, England; d STFC Daresbury Laboratory, Warrington WA4 4AD, England

**Keywords:** *REFMAC*5, refinement

## Abstract

This paper describes various components of the macromolecular crystallographic refinement program *REFMAC*5, which is distributed as part of the *CCP*4 suite. *REFMAC*5 utilizes different likelihood functions depending on the diffraction data employed (amplitudes or intensities), the presence of twinning and the availability of SAD/SIRAS experimental diffraction data. To ensure chemical and structural integrity of the refined model, *REFMAC*5 offers several classes of restraints and choices of model parameterization. Reliable models at resolutions at least as low as 4 Å can be achieved thanks to low-resolution refinement tools such as secondary-structure restraints, restraints to known homologous structures, automatic global and local NCS restraints, ‘jelly-body’ restraints and the use of novel long-range restraints on atomic displacement parameters (ADPs) based on the Kullback–Leibler divergence. *REFMAC*5 additionally offers TLS parameterization and, when high-resolution data are available, fast refinement of anisotropic ADPs. Refinement in the presence of twinning is performed in a fully automated fashion. *REFMAC*5 is a flexible and highly optimized refinement package that is ideally suited for refinement across the entire resolution spectrum encountered in macromolecular crystallography.

## Abstract 2

The general principles behind the macromolecular crystal structure refinement program *REFMAC*5 are described.

## 1. Introduction

As a final step in the process of solving a macromolecular crystal (MX) structure, refinement is carried out to maximize the agreement between the model and the X-ray data. Model parameters that are optimized in the refinement process include atomic coordinates, atomic displacement parameters (ADPs), scale factors and, in the presence of twinning, twin fraction(s). Although refinement procedures are typically designed for the final stages of MX analysis, they are also often used to improve partial models and to calculate the ‘best’ electron-density maps for further model (re)building. Refinement protocols are therefore an essential component of model-building pipelines [*ARP*/*wARP* (Perrakis *et al.*, 1999 ▶), *SOLVE*/*RESOLVE* (Terwilliger, 2003 ▶) and *Buccaneer* (Cowtan, 2006 ▶)] and are of paramount importance in guiding manual model updates using molecular-graphics software [*Coot* (Emsley & Cowtan, 2004 ▶), *O* (Jones *et al.*, 1991 ▶) and *XtalView* (McRee & Israel, 2008 ▶)].

The first software tools for MX refinement appeared in the 1970s. Real-space refinement using torsion-angle parameterization was introduced by Diamond (1971 ▶). This was followed a few years later by reciprocal-space algorithms for the refinement of individual atomic parameters with added energy (Jack & Levitt, 1978 ▶) and restraints (Konnert, 1976 ▶) in order to deliver chemically reasonable models. The energy and restraints approaches differ only in terminology as they use similar information and both can be unified using a Bayesian formalism (Murshudov *et al.*, 1997 ▶). Early programs used the well established statistical technique of least-squares residuals with equal weights on all reflections (Press *et al.*, 1992 ▶), with gradients and second derivatives (if needed) calculated directly. This changed when Fourier methods, which were developed for small-molecule structure refinement (Booth, 1946 ▶; Cochran, 1948 ▶; Cruickshank, 1952 ▶, 1956 ▶), were formalized for macromolecules (Ten Eyck, 1977 ▶; Agarwal, 1978 ▶). The use of the FFT for structure-factor and gradient evaluation (Agarwal, 1978 ▶) sped up calculations dramatically and the refinement of large molecules using relatively modest computers became realistic. Later, the introduction of molecular dynamics (Brünger, 1991 ▶), the generalization of the FFT approach for all space groups (Brünger, 1989 ▶) and the development of a modular approach to refinement programs (Tronrud *et al.*, 1987 ▶) dramatically changed MX solution procedures. Also, the introduction of the very robust and popular small-molecular refinement program *SHELXL* (Sheldrick, 2008 ▶) to the macromolecular community allowed routine analysis of high-resolution MX data, including the refinement of merohedral and non-merohedral twins.

More sophisticated statistical approaches to MX structure refinement started to emerge in the 1990s. Although the basic formulations and most of the necessary probability distributions used in crystallography were developed in the 1950s and 1960s (Luzzati, 1951 ▶; Ramachandran *et al.*, 1963 ▶; Srinivasan & Ramachandran, 1965 ▶; see also Srinivasan & Parthasarathy, 1976 ▶, and references therein), their implementation for MX refinement started in the middle of the 1990s (Pannu & Read, 1996 ▶; Bricogne & Irwin, 1996 ▶; Murshudov *et al.*, 1997 ▶). It should be emphasized that prior to the application of maximum-likelihood (ML) techniques in MX refinement, the importance of advanced statistical approaches to all stages of MX analysis had been advocated by Bricogne (1997 ▶) for two decades. Nowadays, most MX refinement programs offer likelihood targets as an option. Although ML can be very well approximated using the weighted least-squares approach in the very simple case of refinement against structure-factor amplitudes (Murshudov *et al.*, 1997 ▶), ML has the attractive advantage that it is relatively easy (at least theoretically) to generalize for the joint utilization of a variety of sources of observations. For example, it was immediately extended to use experimental phase information (Bricogne, 1997 ▶; Murshudov *et al.*, 1997 ▶; Pannu *et al.*, 1998 ▶). In the last two decades, there have been many developments of likelihood functions towards the exploitation of all available experimental data for refinement, thus increasing the reliability of the refined model in the final stages of refinement and improving the electron density used in model building in the early stages of MX analysis (Bricogne, 1997 ▶; Skubák *et al.*, 2004 ▶, 2009 ▶).

MX crystallography can now take advantage of highly optimized software packages dealing with all of the various stages of structure solution, including refinement. There are several programs available that either are designed to perform refinement or offer refinement as an option. These include *BUSTER*/*TNT* (Blanc *et al.*, 2004 ▶), *CNS* (Brünger *et al.*, 1998 ▶), *MAIN* (Turk, 2008 ▶), *MOPRO* (Guillot *et al.*, 2001 ▶), *phenix.refine* (Adams *et al.*, 2010 ▶), *REFMAC*5 (Murshudov *et al.*, 1997 ▶), *SHELXL* (Sheldrick, 2008 ▶) and *TNT* (Tronrud *et al.*, 1987 ▶). While *MOPRO* was specifically designed for niche ultrahigh-resolution refinement and is able to model deformation density, all of the other programs can deal with a multitude of MX refinement problems and produce high-quality electron-density maps, although with different emphases and strengths.

This contribution describes the various components of the macromolecular crystallographic refinement program *REFMAC*5, which is distributed as part of the *CCP*4 suite (Collaborative Computational Project, Number 4, 1994 ▶). *REFMAC*5 is a flexible and highly optimized refinement package that is ideally suited for refinement across the entire resolution spectrum that is encountered in macromolecular crystallo­graphy.

## 2. Target functions in *REFMAC*5

As in all other refinement programs, the target function minimized in *REFMAC*5 has two components: a component utilizing geometry (or prior knowledge) and a component utilizing experimental X-ray knowledge, 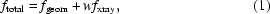
where *f*_total_ is the total target function to be minimized, con­sisting of functions controlling the geometry of the model and the fit of the model parameters to the experimental data, and *w* is a weight between the relative contributions of these two components. In macromolecular crystallography, the weight is traditionally selected by trial and error. *REFMAC*5 offers automatic weighting, which is based on the fact that both components are the natural logarithm of a probability distribution. However, this ‘automatic’ weight may lead to unreasonable deviations from ideal geometry (either too tight or too relaxed) in some cases, as the ideal geometry is difficult to describe statistically. For these cases, the weight parameter may need to be selected manually to produce more reasonable geometry, *e.g.* such that the root-mean-square deviation of the bond lengths from the ideal values is 0.02 Å and at resolutions lower than 3 Å perhaps even smaller.

From a Bayesian viewpoint (O’Hagan, 1994 ▶), these functions have the following probabilistic interpretation (ignoring constants which are irrelevant for minimization purposes): 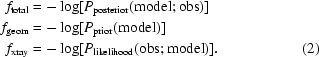
From this point of view, MX refinement is similar to a well known technique in statistical analysis: maximum posterior (MAP) estimation. The model parameters are linked with the experimental data *via f*_xray_, *i.e.* likelihood is a mechanism that controls information flow from the experimental data to the derived model. Consequently, it is important to design a likelihood function that allows optimal information transfer from the data to the derived model. *f*_geom_ ensures that the derived model is consistent with the presumed chemical and structural knowledge. This function plays the role of regularization, reduction of the effective number of parameters and transfer of known information to the new model. If care is not taken, then wrong information may be transferred to the model; removing the effect of such errors may be difficult if possible at all. The design of such functions should be performed using verifiable invariant information and it should be testable and revisable during the refinement and model-building procedures.

Functions dealing with geometry usually depend only on atomic parameters. We are not aware of any function used in crystallography that deals with the prior geometry probability distributions of overall parameters. A possible reason for the lack of interest in (and necessity of) this type of function may be that, despite popular belief, the statistical problem in crystallo­graphy is sufficiently well defined and that the main problems are those of model parameterization and completion.

The existing refinement programs differ in the target functions and optimization techniques used to derive model parameters. Most MX programs use likelihood target functions. However, their form, implementations and parameterizations are different. Therefore, it should not come as a surprise if different programs give (slightly) different results in terms of model parameters, electron-density maps and reliability factors (such as *R* and *R*_free_).

### 2.1. X-ray component

The X-ray likelihood target functions used in *REFMAC*5 are based on a general multivariate probability distribution of *E* observations given *M* model structure factors. This function is derived from a multivariate complex Gaussian distribution of *N* = *E* + *M* structure factors for acentric reflections and from a multivariate real Gaussian distribution for centric reflections and has the following form: 
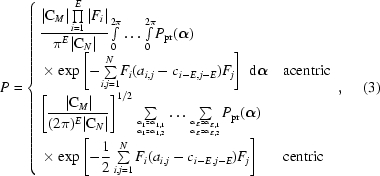
where *P* = *P*(|*F*_1_|, …, |*F_E_*|; *F*_*E*+1_, …, *F_N_*), *F_i_* = |*F_i_*|exp(ια*_i_*}, |*F*_1_|, …, |*F_E_*| denote the observed amplitudes, *F*_*E*+1_, …, *F_N_* are the model structure factors, C*_N_* is the covariance matrix with the elements of its inverse denoted by *a_ij_*, C*_M_* is the bottom right square submatrix of C*_N_* of dimension *M* with the elements of its inverse denoted by *c_ij_*. We define *c_ij_* = 0 for *i* ≤ 0 or *j* ≤ 0. |C*_N_*| and |C*_M_*| are the determinants of matrices C*_N_* and C*_M_*, 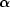 = (α_1_, …, α_*E*_) is the vector of the unknown phases of the observations that need to be integrated and 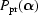 is a probability distribution expressing any prior knowledge about the phases.

In the simplest case of one observation, one model and no prior knowledge about phases, the integral in (3) can be evaluated analytically. In this case, the function follows a Rice distribution (Bricogne & Irwin, 1996 ▶), which is a non-central χ^2^ distribution of |*F*_o_|^2^/Σ and |*F*_o_|^2^/2Σ with non-centrality parameters *D* ^2^|*F*_c_|^2^/Σ and *D* ^2^|*F*_o_|^2^/2Σ with one and two degrees of freedom for centric and acentric reflections, respectively (Stuart & Ord, 2009 ▶), 
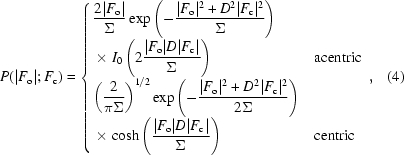
where *D* in its simplest interpretation is 〈cos(Δ*xs*)〉, a Luzzati error parameter (Luzzati, 1952 ▶) expressing errors in the positional parameters of the model, *F*_c_ is the model structure factor, |*F*_o_| is the observed amplitude of the structure factor and Σ is the uncertainty or the second central moment of the distribution. Both Σ and *D* enter the equation as part of the covariance matrices C*_N_* and C*_M_* from (3). Σ is a function of the multiplicity of the Miller indices (∊ factor), experimental uncertainties (σ_o_), model completeness and model errors. For simplicity, the following parameterization is used: 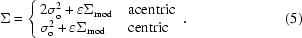
The current version of *REFMAC*5 estimates *D* and Σ_mod_ in resolution bins. Working reflections are used for estimation of *D* and free reflections are used for Σ_mod_ estimation. Although this simple parameterization works in many cases, it may give misleading results for data from crystals with pseudo translation, OD disorder or modulated crystals in general. Currently, there is no satisfactory implementation of the error model to account for these cases.

### 2.2. Incorporation of experimental phase information in model refinement

#### 2.2.1. MLHL likelihood

MLHL likelihood (Bricogne, 1997 ▶; Murshudov *et al.*, 1997 ▶; Pannu *et al.*, 1998 ▶) is based on a special case of the probability distribution (3) where we have one observation, one model and phase information derived from an experiment available as a prior distribution *P*_pr_(α), 
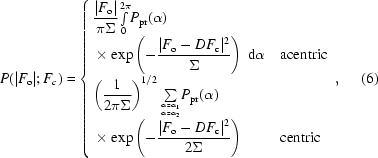
where *F*_o_ = |*F*_o_|exp(ια), *F*_c_ = |*F*_c_|exp(ια_c_), α is the unknown phase of the structure factor and α_1_ and α_2_ are its possible values for a centric reflection. The prior phase probability distribution *P*_pr_(α) is usually represented as a generalized von Mises distribution (Mardia & Jupp, 1999 ▶) and is better known in crystallography as a Hendrickson–Lattman distribution (Hendrickson & Lattman, 1970 ▶), 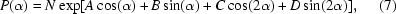
where *A*, *B*, *C* and *D* are coefficients of the Fourier transformation of the logarithm of the phase probability distribution and *N* is the normalization coefficient. The distribution is unimodal when *C* and *D* are zero; otherwise, it is a bimodal distribution that reflects the possible phase uncertainty in experimental phasing. For centric reflections *C* and *D* are zero.

#### 2.2.2. SAD/SIRAS likelihood

The MLHL likelihood is dependent on the reliability and accuracy of the prior distribution *P*_pr_(α). However, the phase distributions after density modification (or even after phasing), which are usually used as *P*_pr_(α), often suffer from inaccurate estimation of the phase errors. Furthermore, MLHL [as well as any other special case of (3) with a non-uniform *P*_pr_(α)] assumes independence of the prior phases from the model phases. These shortcomings can be addressed by using experimental information directly from the experimental data, instead of from the *P*_pr_(α) distributions obtained in previous steps of the structure-solution process. Currently, SAD and SIRAS likelihood functions are implemented in *REFMAC*5.

The SAD probability distribution (Skubák *et al.*, 2004 ▶) is obtained from (3) by setting *E* = 2, *M* = 2, *P*_pr_(α) = constant and |*F*_1_| = |*F*_o_^+^|, |*F*_2_| = |(*F*_o_^−^)*|, *F*_3_ = *F*_c_^+^, *F*_4_ = (*F*_c_^−^)*, where *F* ^+^ and *F* ^−^ are the structure factors of the Friedel pairs. The model structure factors are constructed using the current parameters of the protein, the heavy-atom substructure and the inputted anomalous scattering parameters. Similarly, the SIRAS function (Skubák *et al.*, 2009 ▶) is a special case of (3) with *E* = 3, *M* = 3, *P*_pr_(α) = constant and |*F*_1_| = |*F*_o_ *^N^*|, |*F*_2_| = |*F*_o_^+^|, |*F*_3_| = |(*F*_o_^−^)*|, *F*_4_ = *F*_c_ *^N^, F*_5_ = *F*_c_^+^, *F*_6_ = (*F*_c_^−^)*, where |*F*_1_| and *F*_4_ correspond to the observation and the model of the native crystal, respectively, and |*F*_2_|, |*F*_3_|, *F*_5_ and *F*_6_ refer to the Friedel pair observations and models of the derivative crystal. If any of the *E* observations are symmetrically equivalent, for instance centric Friedel pair intensities, the equation is reduced appropriately so as to only include non-equivalent observations and models.

The incorporation of prior phase information by the refinement function is especially useful in the early and middle stages of model building and at all stages of structure solution at lower resolutions, owing to the improvement in the observation-to-parameter ratio. The refinement of a well resolved high-resolution structure is often best achieved using the simple Rice function.

**Figure 1:**
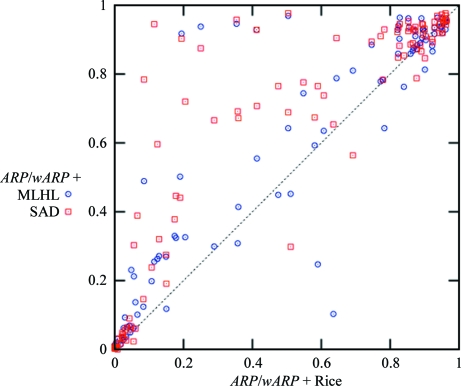
Fraction of the model correctly built by *ARP*/*wARP* v.7.0 iterated with *REFMAC*5 using different target functions. The maps inputted to model building were prepared by *CRANK* (Ness *et al.*, 2004 ▶). The sample consists of 102 data sets described in Skubák *et al.* (2010 ▶).

Fig. 1 ▶ shows the effect of various likelihood functions on automatic model building using *ARP*/*wARP* (Perrakis *et al.*, 1999 ▶).

### 2.3. Twin refinement

The function used for twin refinement is a generalization of the Rice distribution in the presence of a linear relationship between the observed intensities. This function has the form
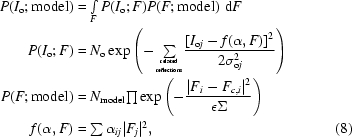
where *N*_o_ and *N*_model_ are normalization coefficients. In the first equation, the first term inside the integral, *P*(*I*_o_; *F*), represents the probability distribution of observations if ‘ideal’ structure factors are known. Here, all reflections that are twinned and that can be grouped together are included. Models representing the data-collection instrument, if available, could be added to this term. The second term, *P*(*F*; model), represents a probability distribution of the ‘ideal’ structure factors should an atomic model be known for a single crystal. Here, all reflections from the asymmetric unit that contribute to the observed ‘twinned’ intensities are included. If the data were to come from more than one crystal or if, for example, SAD should be used simultaneously with twinning, then this term would need to be modified appropriately. *F*_c_ is a function of atomic and overall parameter *D*. Overall parameters also include Σ and twin-fraction parameters. *f* represents the way structure factors from the asymmetric unit contribute to the particular ‘twinned’ intensity. The above formula is more symbolic rather than precise; further details of twin refinement will be published elsewhere.

*REFMAC*5 performs the following preparations before starting refinement against twinned data.

(i) Identify potential (pseudo)merohedral twin operators by analyses of cell/space-group combination using the algorithm developed by Lebedev *et al.* (2006 ▶).
(ii) Calculate *R*_merge_ for each potential twin operator and filter out twin operators for which *R*_merge_ is greater than 0.5 or a user-defined value.
(iii) Estimate twin fractions for the remaining twin domains and filter out those with small twin fractions (the default value is 0.05).
(iv) Make sure that the point group and twin operators form a group. Strictly speaking this stage is not necessary, but it makes bookkeeping easy.
(v) Perform twin refinement using the remaining twin operators. Twin fractions are refined at every cycle.

All integrals necessary for evaluation of the minus log-likelihood function and its derivatives with respect to the structure factors are evaluated using the Laplace approximation (McKay, 2003 ▶).

### 2.4. Modelling bulk-solvent contribution

Typically, a significant part of a macromolecular crystal is occupied by disordered solvent. Accurate modelling of this part of the crystal is still an unsolved problem of MX. The contribution of bulk solvent to structure factors is strongest at low resolution, although its effect at high resolution is still non-negligible.

The absence of good models for disordered solvent may be one of the reasons why *R* factors in MX are significantly higher than those in small-molecular crystallography. For small molecules *R* factors can be around 1%, whereas for MX they are rarely less than 10% and more often around 20% or even higher.

*REFMAC*5 uses two types of bulk (disordered) solvent models. One of them is the so-called Babinet’s bulk-solvent model, which is based on the assumption that the only difference between solvent and protein at low resolution is their scale factor (Tronrud, 1997 ▶). Here, we use a slight modification of the formulation described by Tronrud (1997 ▶) and assume that if protein electron density is convoluted using the Gaussian kernel and multiplied by an appropriate scale factor, then protein and solvent electron densities are equal, 
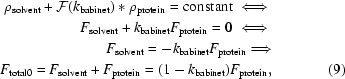
where * denotes convolution, 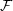 denotes the Fourier transform and *k*_babinet_ = *k*_babinet0_exp(−*B*_babinet_|*s*|^2^/4). Here, we used the convolution theorem, which states that the Fourier transform of the convolution of two functions is the product of their Fourier transforms.

The second bulk-solvent model is derived similarly to that described by Jiang & Brünger (1994 ▶). The basic assumption is that disordered solvent atoms are uniformly distributed over the region of the asymmetric unit that is not occupied by the atoms of the modelled part of the crystal structure. The region of the asymmetric unit occupied by the atomic model is masked out. Any holes inside this mask are removed using a cavity-detection algorithm. A constant value is assigned outside this region and the structure factors *F*_mask_ are calculated using an FFT algorithm. These structure factors, multiplied by appropriate scale factors (estimated during the scaling procedure), are added to those calculated from the atomic model. Additionally, various mask parameters may optionally be optimized.

One should be careful with bulk-solvent corrections, especially when the atomic model is incomplete. This type of bulk-solvent model may result in smeared-out electron density that may reduce the height of electron density in less-ordered and unmodelled parts of the crystal.

The final total structure factors with scale and solvent contributions included take the following form: 
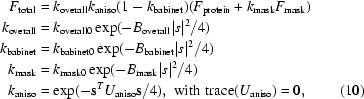
where the *k*s are scale factors, **s** is the reciprocal-space vector, |*s*| is the length of this vector, *U*_aniso_ is the crystallographic anisotropic tensor that obeys crystal symmetry, *F*_mask_ is the contribution from the mask bulk solvent and *F*_protein_ is the contribution from the protein part of the crystal. Usually, either mask or Babinet bulk-solvent correction is used. However, sometimes their combination may provide better statistics (lower *R* factors) than either individually.

The overall parameters of the solvent models, the overall anisotropy and the scale factors are estimated using a least-squares fit of the amplitude of the total structure factors to the observed amplitudes, 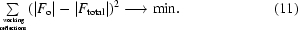
In the case of twin refinement, the following function is used to estimate overall parameters including twin fractions (details of twin refinement will be published elsewhere), 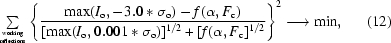
where *f*(α, *F*) is as defined in (8).

Both (11) and (12) are minimized using the Gauss–Newton method with eigenvalue filtering to solve linear equations, which ensures that even very highly correlated parameters can be estimated simultaneously. However, one should be careful in interpretating these parameters as the system is highly correlated.

Once overall parameters such as the scale factors and twin fractions have been estimated, *REFMAC*5 estimates the overall parameters of one of the abovementioned likelihood functions and evaluates the function and its derivatives with respect to the atomic parameters. A general description of this procedure can be found in Steiner *et al.* (2003 ▶).

### 2.5. Geometry component

The function controlling the geometry has several components.

(i) Chemical information about the constituent blocks (*e.g.* amino acids, nucleic acids, ligands) of macromolecules and the covalent links between them.
(ii) Internal consistency of macromolecules (*e.g.* NCS).
(iii) Structural knowledge (known structures, restraints on current interatomic distances, secondary structures).

The first component is used by all programs and has been tabulated in an mmCIF dictionary (Vagin *et al.*, 2004 ▶) now used by several programs, including *REFMAC*5, *phenix.refine* (Adams *et al.*, 2010 ▶) and *Coot* (Emsley & Cowtan, 2004 ▶). The current version of the dictionary contains around 9000 entries and several hundred covalent-link descriptions. Any new entries may be added using one of several programs, including *Sketcher* (Vagin *et al.*, 2004 ▶) from *CCP*4 (Collaborative Computational Project, Number 4, 1994 ▶), *JLigand* (unpublished work), *PRODRG* (Schüttelkopf & van Aalten, 2004 ▶) and *phenix.elbow* (Adams *et al.*, 2010 ▶).

Standard restraints on the covalent structure have the general form
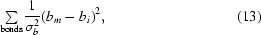
where *b_m_* represents a geometric parameter (*e.g.* bonds, angles, chiralities) calculated from the model and *b_i_* is the ideal value of this particular geometric parameter as tabulated in the dictionary.

Apart from ω (the angle of the peptide bond) and χ (the angles of amino-acid side chains), torsion angles in general are not restrained by default. However, the user can request to restrain a particular torsion angle defined in the dictionary or can define general torsion angles and use them as restraints. In general, it is not clear how to handle the restraint on torsion angles automatically, as these angles may depend on the covalent structure as well as the chemical environment of a particular ligand.

### 2.6. Noncrystallographic symmetry restraints

#### 2.6.1. Automatic NCS definition

Automatic NCS identification in *REFMAC*5 is performed using the following procedure.

(i) Align the sequences of all chains with all chains using the dynamic alignment algorithm (Needleman & Wunsch, 1970 ▶).
(ii) Accept the alignment if the number of aligned residues is more than *k* (default 15) residues and the sequence identity for aligned residues is more than α% (default 80%).
(iii) Calculate the global root-mean-square deviation (r.m.s.d.) using all aligned residues.
(iv) Calculate the average local r.m.s.d. using the formula 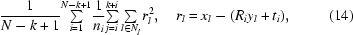
where *N* is the number of aligned residues, *j* indexes the aligned residues, *N_j_* is the number of corresponding atoms in residue *j*, *n*_*j*_ is the number of atoms in the *i*th group, *r_l_* is the vector of differences between corresponding atomic positions and *R_j_* and *t_j_* are the rotation and translation that give the best superposition between atoms in group *i*. To calculate the r.m.s.d., it is not necessary to calculate the rotation and translation operators explicitly or to apply these transformations to atoms. Rather, it is achieved implicitly using Procrustes analysis, as described, for example, in Mardia & Bibby (1979 ▶). When *k* = *N*, the local and global r.m.s.d. coincide.
(v) If the r.m.s.d. is less than β Å (default 2.5 Å), then we consider the chains to be aligned.
(vi) Prepare the list of aligned atoms. If after applying the transformation matrix (calculated using aligned atoms) the neighbours (waters, ligands) of aligned atoms are superimposed, then they are also added to the list of aligned atoms.
(vii) If local NCS is requested, then prepare pairs of corresponding interatomic distances.

Steps (i)–(v) are performed once during each session of refinement. Step (vi) is performed during every cycle of refinement in order to allow conformational changes to occur.

#### 2.6.2. Global NCS

For global NCS restraints, transformation operators (*R_ij_* and *t_ij_*) that optimally superpose all NCS-related molecules are estimated and the following residual is added to the total target function,
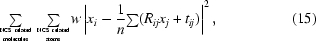
where the weight *w* is a user-controllable parameter. Note that the transformation matrices are estimated using *x_i_* and *x_j_* and thus they are dependent on these parameters. Therefore, in principle the gradient and second-derivative calculations should take this dependence into account, although this dependence is ignored in the current version of *REFMAC*5. Ignoring the contribution of these terms may reduce the rate of convergence, although in practice it does not seem to pose a problem.

#### 2.6.3. Local NCS

**Figure 2:**
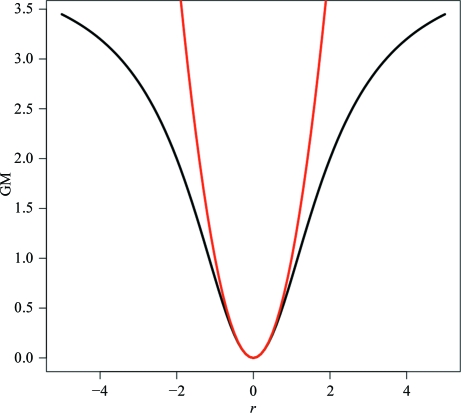
Behaviour of the Geman–McClure function *versus* the quadratic (least-squares) function. For small values of *r* they look similar, whereas for large values of *r* GM is less restrictive than least squares, allowing conformational changes to occur. Black line, GM = *r* ^2^/(1 + σ^2^ *r* ^2^) with σ = 0.5; red line, quadratic function *r* ^2^. This figure was produced using the software package *R* (R Development Core Team, 2007 ▶).

The following function (similar to the implementation in *BUSTER*) is used for local NCS restraints, 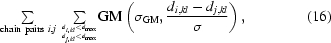
where GM is the Geman–McClure robust estimator function (Geman & McClure, 1987 ▶), which can be written
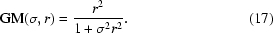
Fig. 2 ▶ shows that for small values of *r* this function is similar to the usual least-squares function. However, it behaves differently for large *r*: least-square residuals do not allow conformational changes to occur, whereas this type of function is more tolerant to such changes.

#### 2.6.4. External structure restraints

The interatomic distances within the structure being analysed may be similar to a known (deposited) structure, particularly in localized regions. In cases where it makes sense, this information can be exploited in order to aid the refinement of the target structure. In doing so, the target structure is pulled towards the con­formation adopted by the known structure. The mechanism for generic external restraints described by Mooij *et al.* (2009 ▶) is used for external structure restraints.

In our implementation, structural information from external known structures is utilized by applying restraints to the distances between atom pairs based on a presumed atomic correspondence between the two structures. The following function is used for external structure restraints, 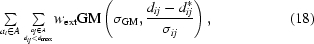
where the atoms *a_i_* belong to the set *A* of atoms for which a correspondence is known, *d_ij_* is the distance between the positions of atoms *a_i_* and *a_j_*, 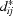 is the corresponding distance in the known structure, σ*_ij_* is the estimated standard deviation of *d_ij_* about 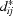 and *d*_max_ ensures that atom pairs are only restrained within localized regions, allowing insensitivity to global conformational changes. External structure restraints should be weighted differently to the other geometry com­ponents in order to allow the restraint strength to be separately specified. Consequently, a weight *w*_ext_ is applied, which should be appropriately chosen depending on the data quality and resolution, the structural similarity between the external known structure and the target, and the choice of *d*_max_. The Geman–McClure function with sensitivity parameter σ_GM_ is used to increase robustness to outliers, as with the local NCS restraints.

Prior information from the external known structure(s) is generated using the software tool *PROSMART*. Specifically, this includes the atomic correspondence *A*, distances 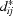, standard deviations σ*_ij_* and the distance cutoff *d*_max_.

Potential sources of prior structural information include different conformations of the target chain (such as those that may result from using different crystallization conditions or in a different binding state) as well as those from homologous or structurally similar proteins. It is possible to use multiple known structures as prior information. The combination of this information results in modified values of 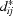 and σ*_ij_* as appropriate. This allows a structure to be refined utilizing information from a whole class of similar structures, rather than just a single source. Furthermore, it opens up the future possibility for multi-crystal co-refinement.

The employed formalism also allows the application of atomic distance restraints to secondary-structure elements (and, in principle, other motifs). Consequently, external restraints may be applied without requiring the prior identification of known structures similar to the target. This is intended to help to refine such motifs towards the expected/presumed local conformation.

This technique has been found to be particularly useful for low-resolution crystals and in cases where the target structure is unable to be refined to a satisfactory level. When used appropriately, external structure restraints should increase refinement reliability. Consequently, the difference between the *R* and *R*_free_ values is expected to decrease in successful cases.

**Figure 3:**
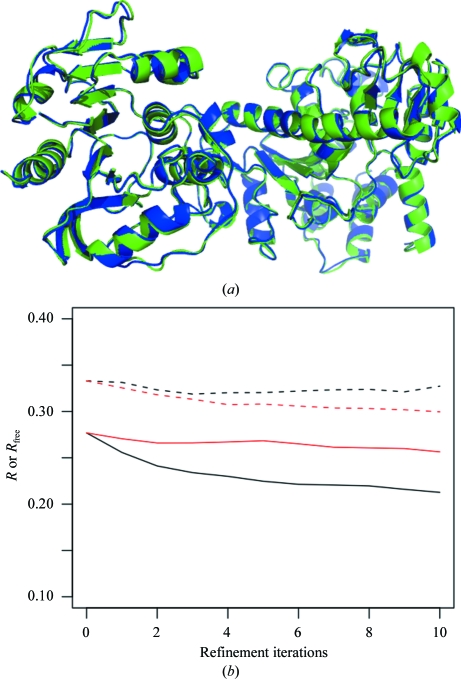
Superposition of the structures of bluetongue virus VP4 enzyme with PDB entries 2jha (green) and 2jhp (blue) (Sutton *et al.*, 2007 ▶), which were solved at 3.4 and 2.5 Å, respectively. The graph shows the resultant *R* (solid) and *R*_free_ (dashed) values from ten iterations of refinement of the low-resolution structure 2jha. Results are shown with (red) and without (black) external restraints, using 2jhp as prior information. This figure was produced using *PROSMART* to superpose the structures, *PyMOL* (DeLano, 2002 ▶) to display the structures and the software package *R* (R Development Core Team, 2007 ▶) to generate the graph.

Fig. 3 ▶ shows the refinement statistics resulting from using external restraints to refine a low-resolution bluetongue virus VP4 enzyme (Sutton *et al.*, 2007 ▶). A sequence-identical structure solved at a higher resolution is used as prior information. Refinement statistics are compared after ten refinement cycles with and without using external restraints. Using the external restraints results in a 2.8% improvement in *R*_free_. Furthermore, the difference between the *R* and *R*_free_ values is reduced from 11.5 to 4.3%, suggesting greatly increased refinement reliability.

#### 2.6.5. ‘Jelly-body’ restraints

The ratio of the number of observations to the number of adjustable parameters is very small at low resolution. Even after accounting for chemical restraints, this ratio stays very small and refinement in such cases is usually unstable. The danger of overfitting is very high; this is reflected in large differences between the *R* and *R*_free_ values. External structure restraints and the use of experimental phase information (described above) provide ways of dealing with this problem. Unfortunately, it is not always possible to find similar structures refined at high resolution (or at least ones that result in a sufficiently successful improvement in refinement statistics) and experimental phase information is not always available or sufficient. Fortunately, statistical techniques exist to deal with this type of problem. Such techniques include ridge regression (Stuart *et al.*, 2009 ▶), the lasso estimation procedure (Tibshirani, 1997 ▶) and Bayesian estimation with prior knowledge of parameters (O’Hagan, 1994 ▶).

*REFMAC*5 has a regularization function in interatomic distance space that has the form
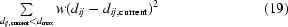
for pairs of atoms *i*, *j* from the same chain, with maximum radius *d*_max_, which can be controlled (default 4.25 Å). Note that this term does not contribute to the value of the function or its gradient; it only changes the second derivative, thus changing the search direction. It should be noted that a similar technique has been implemented in *CNS* (Schröder *et al.*, 2010 ▶).

Note that if all interatomic distances were constrained, then individual atomic refinement would become rigid-body refinement. The effect of ‘jelly-body’ restraints is the implicit parameterization between the rigid body and individual atoms. This technique has strong similarity to elastic network model calculations (Trion, 1996 ▶). This simple formula has been found to work surprisingly well.

#### 2.6.6. Atomic displacement parameter restraints

Unlike positional parameters, where prior knowledge can be designed using basic knowledge of the chemistry of the building blocks of macromolecules and analysis of high-resolution structures, it is not obvious how to design restraints for atomic displace­ment parameters (ADPs). Ideally, restraints should reflect the geometry of the molecules as well as their overall mobility. Various programs use various restraints (Sheldrick, 2008 ▶; Adams *et al.*, 2010 ▶; Konnert & Hendrickson, 1980 ▶; Murshudov *et al.*, 1997 ▶). In the new version of *REFMAC*5, restraints on ADPs are based on the distances between distributions. If we assume that atoms are represented as Gaussian distributions, then we are able to design restraints based on the distance between such distributions.

For a given two distributions in three-dimensional space *P*(*x*) and *Q*(*x*), the symmetrized Kullback–Liebler (KL) divergence (McKay, 2003 ▶) is defined as follows: 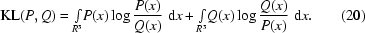
It can be verified that the symmetrized KL divergence satisfies the conditions of a metric distance in the space of distributions. The KL divergence can also be represented as follows: 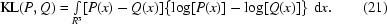
This distance changes more smoothly than the *L*_2_ distance between functions and seems to be a useful criterion for the design of approximate probability distributions (McKay, 2003 ▶; O’Hagan, 1994 ▶).

When both distributions are Gaussian with mean zero, this distance has an elegant form. Assume that both atoms have Gaussian distribution: 
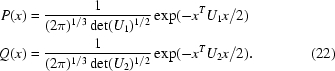
In this case, the KL divergence becomes 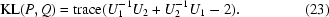
In the case of isotropic ADPs, KL has an even simpler form: 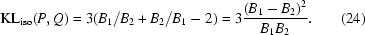

                  *REFMAC*5 uses restraints based on the KL divergence: 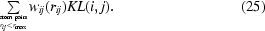
The summation is over all atom pairs with distance less than *r*_max_. The weights depend on the nature of the bonds as well as on the distance between the atoms. If atoms are bonded or angle-related then the weight is larger. However, the weight is smaller if the atoms are not related by covalent bonds. Moreover, if the distance between the atoms is more than 3 Å then the weight decreases as follows: 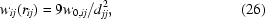
where *w*_0,*ij*_ is the weight for nonbonded atoms that are closer than 3 Å to each other.

#### 2.6.7. Rigid-bond restraints

For anisotropic atoms there are so-called rigid-bond restraints, based on the idea of rigid-bond tests of anisotropic atoms (Hirshfeld, 1976 ▶). The idea is that projections of *U* values on the bond vector joining two atoms should be similar. In other words, if two atoms are bonded then an oscillation across the bond is more likely than an independent oscillation along the bond. Atoms oscillate along the bond in a concerted fashion.

Rigid-bond restraints are designed as follows. Let us assume that two atoms have positions *x*_1_ and *x*_2_ and their corresponding ADPs are *U*_1_ and *U*_2_;  the unit vector joining these atoms is then calculated, 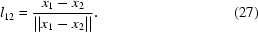
The projections of corresponding *U* values on this vector are then calculated as
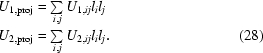
Now, using these projections, the KL divergence is formed for all pairs and added to the target function: 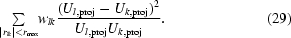
Again, the weights depend on the nature of the bonds between the atoms and the distances between them. Note that if the ADPs of both bonded atoms are isotropic then the rigid-bond restraint is equivalent to the above-described KL restraint.

#### 2.6.8. Sphericity restraints

To avoid atoms exploding and becoming too elliptical or, even worse, non-elliptical, *REFMAC*5 uses restraints on sphericity. It is a simple restraint: an isotropic equivalent of the anisotropic tensor, 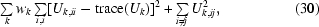
where *k* indexes the anisotropic atoms, *i*, *j* are components of the anisotropic tensor and *w_k_* are weights for this particular type of restraint. The weights depend on the number of other restraints (KL, rigid bond) on this atom. Atoms that have fewer restraints have stronger weights on sphericity, since these atoms are more likely to be unstable.

It should be noted that similar restraints on ADPs are used in several other refinement programs (Sheldrick, 2008 ▶; Adams *et al.*, 2010 ▶).

## 3. Parameterization

### 3.1. General parameters

*REFMAC*5 uses the standard parameterization of molecules in terms of atomic coordinates and isotropic/anisotropic atomic displacement parameters. The refinement of these parameters is performed using an FFT formulation for gradients and approximations for second derivatives. Details of these formulations have been published elsewhere (Murshudov *et al.*, 1997 ▶, 1999 ▶; Steiner *et al.*, 2003 ▶). Once the gradients and approximate second derivatives have been calculated for these parameters, they are used to calculate the derivatives of derived parameters. Derived parameters include those for rigid-body and TLS refinement.

### 3.2. Rigid body

Rigid-body parameterization is achieved as follows. For each rigid group, transformation operators are defined and new positions are calculated from the starting positions using the formula
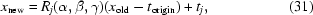
where *R_j_* is the rotation matrix, *t*_origin_ is the centre of mass of the rigid group and *t_j_* is the translational component of the transformation. The *x*_old_ are the starting coordinates of the atoms and *x*_new_ are their positions after application of the transformation operators. There are six parameters per rigid group, defining the rotation matrix and the translational component. At each cycle of refinement, an eigenvalue-filtering technique is used to avoid potential singularities arising from the shape of the rigid groups. It should be noted that no terms between rigid groups are calculated for the approximate second-derivative matrix. For large rigid groups this does not pose much of a problem. However, for many small rigid groups it may slow down convergence substantially. In any case, it is not recommended to divide molecules into very small rigid groups. For these cases, ‘jelly-body’ refinement should produce better results.

Once derivatives with respect to the positional parameters have been calculated, those for rigid-body parameters are calculated using the chain rule. The current version of *REFMAC*5 uses an Euler angle parameterization.

### 3.3. TLS

Atomic displacement parameters describe the spread of atomic positions and can be derived from the Fourier transform of a Gaussian probability distribution function for the atomic centre. The atomic displacement parameters are an important part of the model. Traditionally, a single parameter describing isotropic displacements has been used, namely the *B* factor. However, it is well known that atomic displacements are likely to be anisotropic owing to directional bonding and at high resolutions the six parameters per atom of a fully anisotropic model can be refined. TLS refinement is a way of modelling anisotropic displacements using only a few parameters, so that the method can be used at medium and low resolutions. The TLS model was originally proposed for small-molecule crystallography (Schomaker & Trueblood, 1968 ▶) and was incorporated into *REFMAC*5 almost ten years ago (Winn *et al.*, 2001 ▶).

The idea behind TLS is to suppose that groups of atoms move as rigid bodies and to constrain the anisotropic displace­ment parameters of these atoms accordingly. The rigid-body motion is described by translation (T), libration (L) and screw (S) tensors, using a total of 20 parameters for each rigid body. Given values for these 20 parameters, anisotropic displacement parameters can be derived for each atom in the group (and this relationship also allows one to calculate derivatives *via* the chain rule). Usually, an extra isotropic displacement parameter (the residual *B* factor) is refined for each atom in addition to the TLS contribution. The sum of these two con­tributions can be output using the supplementary program *TLSANL* (Howlin *et al.*, 1993 ▶) or optionally directly from *REFMAC*5.

TLS groups need to be chosen before refinement and constitute part of the definition of the model for the macromolecule. Groups of atoms should conform to the idea that they move as a quasi-rigid body. Often the choice of one group per chain suffices (or at least serves as a reference calculation) and this is the default in *REFMAC*5. More detailed choices can be made using methods such as *TLSMD* (Painter & Merritt, 2006 ▶). By default, *REFMAC*5 also includes waters in the first hydration shell, which it seems reasonable to assume move in concert with the protein chain.

**Figure 4:**
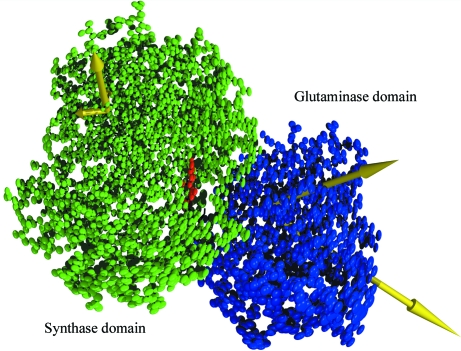
TLS refinement of glucosamine-6-phosphate synthase (Mouilleron & Golinelli-Pimpaneau, 2007 ▶). The results for chain *C* are shown, which is separated into two TLS groups. Thermal ellipsoids derived from the TLS refinement are shown for the two groups. Those in red correspond to the ligand Fru6P which is included in the TLS group for the synthase domain. The yellow arrows show the principal axes of the libration tensor for each TLS group. Inclusion of TLS parameters led to a reduction in *R* and *R*_free_ of 3.4% and 3.8%, respectively, and could be related to the biological function. The principal axis of the libration tensor was calculated using *TLSANL* (Howlin *et al.*, 1993 ▶) and the figure was prepared using *CCP*4*mg* (Potterton *et al.*, 2004 ▶).

Fig. 4 ▶ shows the effect of TLS refinement and orientation of libration tensors. In this case, TLS refinement improves *R*/*R*_free_ and the derived libration tensors make biological sense.

## 4. Optimization

*REFMAC*5 uses the Gauss–Newton method for optimization. For an elegant and comprehensive review on optimization techniques, see Nocedal & Wright (1999 ▶). In this method, the exact second derivative is not calculated, but rather approximated to make sure it is always non-negative. Once derivatives or approximations have been calculated, the following linear equation is built, 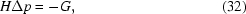
where *H* is the approximate second derivative and *G* is the gradient vector. The contribution of most of the geometrical terms are calculated using algorithms designed for quadratic optimization or least-squares fitting (Press *et al.*, 1992 ▶). To calculate the contribution from the Geman–McClure terms, the following approximation is used (Huber & Ronchetti, 2009 ▶), 
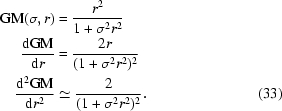
This approximation ensures that *H* stays non-negative and consequently directions calculated as a result of the solution of (32) point towards a reduction of the total function.

The contribution of the X-ray term to the gradient is calculated using FFT algorithms (Murshudov *et al.*, 1997 ▶). The Fisher information matrix, as described by Steiner *et al.* (2003 ▶), is used to calculate the contribution of the likelihood functions to the matrix *H*. Tests have demonstrated that using the diagonal elements of the Fisher information matrix and both diagonal and nondiagonal elements of the geometry terms results in a more stable refinement.

Once all of the terms contributing to *H* and *G* have been calculated, the linear equation (32) is solved using preconditioned conjugate-gradient methods (Nocedal & Wright, 1999 ▶; Tronrud, 1992 ▶). A diagonal matrix formed by the diagonal elements of *H* is used as a preconditioner. This brings parameters with different overall scales (positional and *B* values) onto the same scale and controlling convergence becomes easier.

If the conjugate-gradient procedure does not converge in *N*_maxiter_ cycles (the default is 1000), then the diagonal terms of the *H* matrix are increased. Thus, if the matrix is not positive then ridge regression is activated. In the presence of a potential (near-) singularity, *REFMAC*5 uses the following procedure to solve the linear equation.

(i) Define and use preconditioner. At this stage, *H* and *G* are modified. Define the new matrix by *H*_1_ and vector by *G*_1_.
(ii) Set γ = 0.
(iii) Define a new matrix: *H*_2_ = *H*_1_ + γ*I*, where *I* is the identity matrix.
(iv) Solve the equation *H*_2_ *p* = −*G*_1_ using the conjugate-gradient method for linear equations for sparse and positive-definite matrices (Press *et al.*, 1992 ▶). If convergence was achieved in less than *N*_maxiter_ iterations, then proceed to the next step. Otherwise, increase γ and go to step (iii).
(v) Decondition the matrix, gradient and shift vectors.
(vi) Apply shifts to the atomic parameters, making sure that the ADPs are positive.
(vii) Calculate the value of the total function.
(viii) If the value of the total function is less than the previous value, then proceed to the next step. Otherwise, reduce the shifts and repeat steps (vi)–(viii).
(ix) Finish the refinement cycle.

After application of the shifts, the next cycle of refinement starts.

## 5. Conclusions

Refinement is an important step in macromolecular crystal structure elucidation. It is used as a final step in structure solution, as well as as an intermediate step to improve models and obtain improved electron density to facilitate further model rebuilding.

*REFMAC*5 is one of the refinement programs that incorporates various tools to deal with some crystal peculiarities, low-resolution MX structure refinement and high-resolution refinement. There are also tabulated dictionaries of the constituent blocks of macromolecules, cofactors and ligands. The number of dictionary elements now exceeds 9000. There are also tools to deal with new ligands and covalent modifications of ligands and/or proteins.

Low-resolution MX structure analysis is still a challenging task. There are several outstanding problems that need to be dealt with before we can claim that low-resolution MX analysis is complete. Statistics, image processing and computer science provide general methods for these and related problems. Unfortunately, these techniques cannot be directly applied to MX structure analysis, either because of the huge computer resources needed or because the assumptions used are not applicable to MX.

In our opinion, the problems of state-of-the-art MX analysis that need urgent attention include the following.

(i) Reparameterization depending on the quality and the amount of experimental data. Some tools implemented in *REFMAC*5 allow partial dealing with this problem. These tools include (*a*) restraining against known homologous structures, (*b*) ‘jelly-body’ restraints or refinement along implicit normal modes, (*c*) long-range ADP restraints based on KL divergence, (*d*) automatic local and global NCS restraints and (*e*) experimental phase-information restraints. However, low-resolution refinement and model (re)building is still not as automatic as for high-resolution structures.
(ii) Statistical methods for peculiar crystals with low signal-to-noise ratio. Some of the implemented tools, such as likelihood-based twin refinement and SAD/SIRAS refinement, help in the analysis of some of the data produced by such crystals. The analysis of data from such peculiar crystals as OD disorder with or without twinning, multiple cells, translocational disorder or modulated crystals in general remains problematic.
(iii) Another important problem is that of limited and noisy data. As a result of resolution cutoff (owing to the survival time of the crystal under X-ray irradiation or otherwise), the resultant electron density usually exhibits noise owing to series termination. If the resolution that the crystal actually diffracts to is the same as the resolution of the data, then series termination is not very serious as the signal dies out towards the limit of the resolution. However, in these cases the electron density becomes blurred, reflecting high mobility of the molecules or crystal disorder. When map sharpening is used, the signal is amplified and series termination becomes a serious problem. To reduce noise, it is necessary to work with the full Fourier transformation. In other words, resolution extension and the prediction of missing reflections becomes an important problem. The dramatic effect of such an approach for density modification at high resolution has been demonstrated by Altomare *et al.* (2008 ▶) and Sheldrick (2008 ▶). The direct replacement of missing reflections by calculated ones necessarily introduces bias towards model errors and may mask real signal. To avoid this, it is necessary to integrate over the errors in the model parameters (coordinates, *B* values, scale values and twin fractions). However, since the number of parameters is very large (sometimes exceeding 1 000 000), integration using available numerical techniques is not feasible.
(iv) Error estimation. Despite the advances in MX, there have been few attempts to evaluate errors in the estimated parameters. Works attempting to deal with this problem are few and far between (Sheldrick, 2008 ▶). To complete MX structure analysis, it is necessary to develop and implement techniques for error estimation. If this is achieved, then incorrect structures could be eliminated while analysing the MX data and building the model. One of the promising approaches to this problem is the Gauss–Markov random field sampling technique (Hue & Held, 2005 ▶) using the (approximate) second derivative as a field-defining matrix.
(v) Multicrystal refinement with the simultaneous multicrystal averaging of isomorphous or non-isomorphous crystals is one of the important directions for low-resolution refinement. If it is dealt with properly then the number of structures analysed at low resolution should increase substantially.

Further improvement may consist of a combination of various experimental techniques. For example, the simultaneous treatment of electron-microscopy (EM) and MX data could increase the reliability of EM models and put MX models in the context of larger biological systems.

The direct use of unmerged data is another direction in which refinement procedures could be developed. If this were achieved, then several long-standing problems could be easier to deal with. Two such problems are the following. (i) In general, the space group of a crystal should be considered as an adjustable parameter. If unmerged data are used, then space-group assumptions could be tested after every few sessions of refinement and model building. (ii) Dealing with the processes in the crystal during data collection requires unmerged data. One of the best-known such problems is radiation damage.
